# Management of ulcerative colitis

**DOI:** 10.1136/archdischild-2014-307218

**Published:** 2015-11-09

**Authors:** John M Fell, Rafeeq Muhammed, Chris Spray, Kay Crook, Richard K Russell, BSPGHAN IBD working group

**Affiliations:** 1 Department of Paediatric Gastroenterology, Chelsea and Westminster Hospital NHS Foundation Trust, London, UK; 2 Department of Paediatric Gastroenterology, Birmingham Children's Hospital NHS Foundation Trust, Birmingham, UK; 3 Department of Paediatric Gastroenterology, Bristol Royal Hospital for Children, Bristol, UK; 4 Department of Paediatric Gastroenterology, Northwick Park Hospital, London, UK; 5 Department of Paediatric Gastroenterology, The Royal Hospital for Children, Glasgow, UK

**Keywords:** Gastroenterology, Multidisciplinary team-care, Paediatric Surgery

## Abstract

Ulcerative colitis (UC) in children is increasing. The range of treatments available has also increased too but around 1 in 4 children still require surgery to control their disease. An up-to-date understanding of treatments is essential for all clinicians involved in the care of UC patients to ensure appropriate and timely treatment while minimising the risk of complications and side effects.

**Summary points**

- The management of ulcerative colitis (UC) requires an understanding of the available medications and their side effects.
- The Paediatric Ulcerative Colitis Activity Index is a useful tool for recording disease severity and its changes over time.
- Paediatricians need to be aware of the risks associated with acute severe colitis and the need for appropriate and timely escalation of medical management and the role of surgery in some cases.
- Recognition and treatment of iron deficiency (and other nutritional deficits) is important for the general well-being of children with UC.

## Introduction

Within the diagnostic label of inflammatory bowel disease (IBD), ulcerative colitis (UC) is typically characterised by diffuse mucosal inflammation, limited to the colon, with confluent inflammation extending proximally from the rectum to varying severity and extent. In children, it is well recognised however, that UC typically affects all or most of the colon (extensive or pan-colitis), whereas adults more often present with localised distal disease (proctitis).^1^ Recently, the paediatric Porto criteria for the diagnosis of IBD have been revised to take account of diagnostic advances, for example, in imaging techniques, and also to help correctly assign patients to the differing IBD subgroups.^2^ In the paediatric age group, up to 10% of cases cannot be assigned to as either UC or Crohn's despite comprehensive assessment. These are classified as IBD unclassified, although in practice, currently, they are most commonly treated along the same lines as UC.

## Diagnostic tests for suspected inflammatory bowel disease

The majority of children with ulcerative colitis (UC) present with the characteristic features of diarrhoea, blood in stools and abdominal pain, which indicate a colitic process. Blood tests (full blood count, erythrocyte sedimentation rate, C reactive protein, liver function tests and albumin) will show abnormalities in around 80% of cases in inflammatory bowel disease (IBD), and thus, form part of the initial assessment of suspected cases.^3^ Faecal tests such as calprotectin are useful adjuncts to these blood tests in identifying inflammation, but levels can be elevated by causes apart from IBD.^4^

In cases with suspected IBD, following exclusion of an infective cause, both upper and lower gastrointestinal (GI) (ileocolonic) endoscopy are required. Imaging of the small bowel is also recommended.^2^ With the more widespread availability of magnetic resonance enterography this is now the modality of choice, when available, for differentiating UC from Crohn's disease (CD). It is important to exclude *Clostridium difficile* (C. diff) infection in the differential diagnosis of IBD initially and exclude C. diff infection as the cause of a disease ‘flare’. Despite recommendations to screen for C. diff in these situations, it can often be difficult in practice as this test has not been historically widely performed in children.

Extraintestinal manifestations (EIMs) can occur in 6%–17% of patients with UC at diagnosis, and can increase with disease evolution to nearly 50%. EIMs can affect joints (arthritis), liver (primary sclerosing cholangitis, which affects 1.6% of cases of paediatric IBD at 10 years, autoimmune hepatitis), skin (pyoderma gangrenosum) and eyes (uveitis).^5^ Sclerosing cholangitis can be associated with progressive liver disease and cholangicarcinoma.^6^ Furthermore, there is an increased risk of colonic dysplasia in sclerosing cholangitis associated UC.^7^ Thus, in these cases, surveillance colonoscopy will need to be initiated earlier (possibly into the paediatric age range depending on age at diagnosis) and followed more frequently.

## Clinical scoring with the Paediatric Ulcerative Colitis Activity Index

The Paediatric Ulcerative Colitis Activity Index (PUCAI) (table 1) has been devised as a clinical score of disease severity, which should now be used by all paediatricians looking after patients with UC to objectively assess their disease. By differentially weighting the severity of the main clinical features of UC (rectal bleeding, stool frequency and consistency, abdominal pain and general activity levels), a score between 0 and 85 can be derived.^8^ This can then be used to record disease severity at a point in time plus measure the response to therapy. A PUCAI score of <10 denotes remission, 10–30 denotes mild disease, 35–60 denotes moderate disease, while a score of ≥65 represents acute severe colitis (ASC), which is a medical emergency, and thus, recommended management follows a distinct pathway.

**Table 1:** Paediatric Ulcerative Colitis Activity Index (PUCAI)^8^

Item	Points
(1) Abdominal pain	
No pain	0
Pain can be ignored	5
Pain cannot be ignored	10
(2) Rectal bleeding	
None	0
Small amount only, in <50% of stools	10
Small amount with most stools	20
Large amount (>50% of stool content)	30
(3) Stool consistency of most stools	
Formed	0
Partially formed	5
Completely unformed	10
(4) Number of stools per 24 h	
0–2	0
3–5	5
6–8	10
>8	15
(5) Nocturnal stools (any episode causing wakening)	
No	0
Yes	10
(6) Activity level	
No limitation of activity	0
Occasional limitation of activity	5
Severely restricted activity	10
PUCAI total (0–85)	

PUCAI score <10, remission; 10–34, mild; 35–64, moderate; ≥65, severe.

The clinical features of severe UC (ASC) include the typical symptoms of bloody diarrhoea (usually ≥6 a day with nocturnal defaecation), abdominal pain and reduced activity. In addition, there may be further systemic symptoms of vomiting, tachycardia and fever, which can be accompanied by life-threatening toxic dilation of the colon. *The recognition of ASC is of vital importance, and should be considered a medical emergency* with immediate referral to a paediatric gastroenterology unit with paediatric surgical support being essential. Subsequent repeat PUCAI scoring is very helpful in monitoring disease activity and the response to therapy (see below).

### Medical management of UC

The treatment recommendations are based on the guidelines produced by the National Institute for Health and Care Excellence and the joint guidelines produced by the European Crohn's and Colitis Organisation (ECCO) and European Society for Paediatric Gastroenterology Hepatology and Nutrition (ESPGHAN).^9–11^ These guidelines provide very useful and comprehensive algorithms, which cover most common clinical eventualities.

Most patients with UC can be treated on an outpatient basis, but with hospitalisation necessary for ASC. The main aim of treatment of UC in children is to achieve maximum possible symptomatic control with minimal side effects, while allowing children to function as normally as possible. The target for treatment increasingly is also looking at intestinal healing beyond simple symptomatic control to try and reduce the risk of long-term complications and surgery.^1^ ^2^ Treatments can be broadly considered as those used to induce remission (at diagnosis or for a subsequent flare), such as 5-aminosalicylic acid (5-ASA) agents, corticosteroids and biologics, and those used for long-term maintenance of remission such as 5-ASA agents, biologics and thiopurines.

## 5-ASA agents

The mainstay of therapy for mild-to-moderate UC is sulfasalazine and other 5-ASA agents (eg, mesalazine). These agents are effective in inducing remission and also in maintaining remission for patients with mild and some with moderate disease.^13^ 5-ASA preparations are generally preferred to sulfasalazine due to a superior side effect profile combined with similar efficacy. However, in younger children (preschool), the absence of a liquid preparation for 5-ASA means that sulfasalazine will often be used. 5-ASA preparations are available as granules, and are, thus, useful for those unable to swallow tablets such as children of primary school age. A summary of available preparations and their licencing status is given in table 2.

**Table 2:** Commonly used mesalazine preparations

Drug	Formulation	Optimal drug release pH	Site of drug release
Asacol	Enteric coated with Eudragit S	pH-dependent delayed release (>7)	Terminal ileum and colon
Ipocol	Enteric coated with Eudragit S	>7	Terminal ileum and colon
Octasa	Enteric coated with Eudragit S	pH-dependent delayed release (>7)	Terminal ileum and colon
Pentasa	Ethyl cellulose coated microgranules	Diffusion through semipermeable membrane (enteral pH)	Duodenum to colon
Salofalk	Tablets: enteric coated with Eudragit L; Granules: Eudragit L plus matrix granule structure	pH-dependent delayed release (>6) Granules have extra delayed release	Terminal ileum and colon

*Licensed use*: Asacol (all preparations) and Salofalk enema are not licensed for use in children under 18 years; Salofalk suppositories, Pentasa tablets and suppositories are not licensed for use in children under 15 years; Pentasa granules and Salofalk granules not licensed for use in children under 6 years.

Oral mesalazine and sulfasalazine are usually given in divided doses, but there is evidence from adult studies that appropriately formulated 5-ASA is equally effective when given once daily.^14^ The generally benign side-effect profile of 5-ASAs has also resulted in a tendency towards rather higher dosing. The maintenance dose should be similar to the dose used for induction therapy, although the dose can be reduced after a period of sustained remission. Topical 5-ASA (suppositories or enemas) are effective in mild-to-moderate distal UC, and usually combining oral and enema therapy is more effective than either treatment alone for extensive as well as distal UC.^15^ Lack of response to mesalazine within 2–4 weeks is an indication to consider treatment with corticosteroids.

## Corticosteroids

The majority of patients with moderate-to-severe UC achieve remission on treatment with oral steroids. However, steroid treatment is not advised for maintenance of remission. The dose of prednisone/prednisolone should be 1 mg/kg up to 40 mg once daily in most children. A single total dose in the morning is advisable to reduce potential harmful suppression of growth. There are no universally accepted steroid-tapering protocols, but the ECCO paediatric UC guideline provides a practical 10-week reduction scheme (table 3).^11^ Steroid dependency is defined as response or remission with corticosteroid treatment, but recurrence of symptoms when the dose is lowered or within 3 months following complete taper, or if steroids cannot be stopped within 14–16 weeks. Children with severe UC will need intravenous steroid treatment (see below). Steroid dependency can be avoided by escalating maintenance therapy. For distal disease, this typically means a 5-ASA rather than steroid enema, whereas for more extensive colitis, oral beclomethasone dipropionate, a corticosteroid with topical action, can be considered.^16^

**Table 3:** Steroid tapering table

Week 1	Week 2	Week 3	Week 4	Week 5	Week 6	Week 7	Week 8	Week 9	Week 10	Week 11
60*	50	40	35	30	25	20	15	10	5	0
50*	40	40	35	30	25	20	15	10	5	0
45*	40	40	35	30	25	20	15	10	5	0
40	40	30	30	25	25	20	15	10	5	0
35	35	30	30	25	20	15	15	10	5	0
30	30	30	25	20	15	15	10	10	5	0
25	25	25	20	20	15	15	10	5	5	0
20	20	20	15	15	12.5	10	7.5	5	2.5	0
15	15	15	12.5	10	10	7.5	7.5	5	2.5	0

Prednisolone tapering plan. The daily milligram (mg) dose is changed weekly (week) according to this plan.^11^

*The initial recommended prednisolone dose is 1 mg/kg/day maximum 40 mg/day except for cases discharged following acute severe colitis where higher doses up to 60 mg/day could be used.

## Thiopurines (azathioprine and 6-mercaptopurine)

Azathioprine and 6-mercaptopurine (6-MP) are purine analogues commonly used in the treatment of steroid-dependent IBD and in children with very severe initial presentation of UC. The therapeutic effect of thiopurines may take up to 10–14 weeks after the start of treatment. The recommended dose is 2.5 mg/kg of azathioprine and 1–1.5 mg/kg of 6-MP, in a single daily dose. The most recent recommendation is to start on the maximum dose of thiopurine with no need to ‘build’ up the dose as was practiced historically.^11^ Common side effects include headache, rash and GI disturbance. Some patients experience an influenza-like illness, and pancreatitis occurs in between 1% and 4% of patients. Patients should be informed of the benefits of treatments and how they are balanced against the side-effect profile, including a small, but increased, risk of malignancy most notably lymphoma.

**Figure 1:**
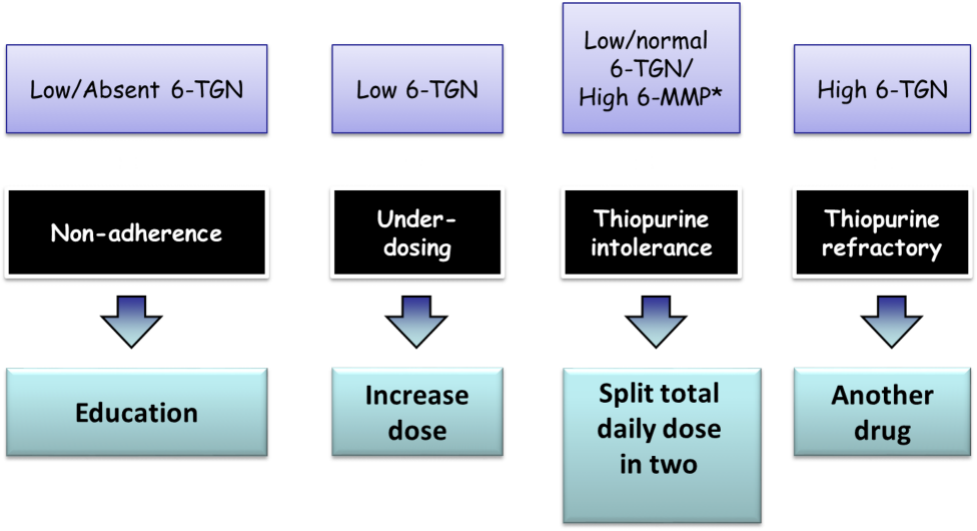
Suggested pathway for reacting to thiopurine metabolite results. This algorithm is designed predominantly to help patients with active disease as we assume patients in remission would not routinely have levels measured. *The majority of patients with a raised 6-methyl mercaptopurine (6-MMP) need no action taken unless there is evidence of a transaminitis. The established ranges for 6-thioguanine (6-TGN) levels and 6-MMP commonly used in the UK are 235–450 pmol (Purine Research Laboratory at St Thomas, London). Low and high in the figure refer to values lying outside these ranges. Allopurinol and dose reduction is used rarely in paediatric practice to help with thiopurine toleration, but should only be considered in specialist units with appropriate experience and monitoring arrangements in place.

The determination of thiopurines methyltransferase (TPMT) genotype or phenotype is recommended to identify patients at greater risk of early profound myelosuppression due to homozygote mutant/very low TPMT activity status. It is the authors’ practice to half the recommended dose in heterozygous patients or in those with low (but not very low) enzyme activity. Thiopurines should not be used in children who are homozygous for TPMT polymorphisms or those with extremely low TPMT activity. Myelosuppression may still occur in the presence of normal TPMT activity, and therefore, regular monitoring of blood counts and liver tests are recommended in all cases. Measurement of thiopurine metabolites, 6-methyl mercaptopurine and 6-thioguanine is helpful to assess compliance and optimisation of the therapy. A suggested practice-based algorithm for using these metabolites is shown in figure 1.

## Anti-tumour necrosis factor antibodies

Infliximab, adalimumab and golimumab are the anti-tumour necrosis factor alpha agents available in the UK for the treatment of UC in adults, but the mainstay of paediatric practice is with infliximab only. Golimumab and adalimumab do not have the marketing authorisation in the UK for the treatment of UC in children. Infliximab should be considered for treatment of children with chronically active or steroid-dependent UC, uncontrolled by 5-ASA and thiopurines. Infliximab may also be considered for steroid-refractory disease and acute severe disease. Measuring infliximab levels and antibodies to infliximab can optimise treatment when patients fail to respond to therapy either after initial induction treatment or at follow-up. There is evidence from adult studies that combining infliximab with a thiopurine improves effect in patients with UC, but the efficacy of combination treatment should be weighed against the risk of side effects, including an increased relative risk of lymphoma.^17^

## Compliance (oral and rectal preparations)

Compliance to administering and continuing oral and rectal drugs can be difficult. Care will be taken by the initially prescribing specialist to choose with the patient and caregiver the optimal oral preparation (tablets, granules or suspension). As for rectal preparations, the indicated and best-suited rectal preparation for distal disease (suppository, enema, foam enema) will be evaluated, and patients need to be advised on the application to maximise tolerance and compliance. This is usually best done by an IBD nurse with a good knowledge of all of the specific available products matched with which product will be best suited to the individual patient.

## Antibiotics/probiotics

There is not enough evidence to recommend probiotics or antibiotics for routine use in UC. There is evidence for the probiotic *Escherichia coli* Nissle 1917 for patients with mild UC who are intolerant to 5-ASA or as adjuvant therapy.^18^ In pouchitis (inflammation of the pouch created after a colectomy), a combination probiotic (VSL#3) is useful in reducing the risk of further episodes of pouchitis. More recently, a combination of antibiotics has been used for paediatric patients resistant to other treatments with some promising early results.^19^

## Newer treatment agents

Vedolizumab (humanised monoclonal antibody that specifically recognises the α_4_β_7_ heterodimer and selectively blocks gut lymphocyte trafficking), tofacitinib (oral Janus kinase inhibitor) and etrolizumab (humanised monoclonal antibody targeting the β7 subunit of the heterodimeric integrins α4β7 and αEβ7) have shown promising results in the treatment of adult patients with UC.^20–22^ Paediatric trials of these agents are awaited. Faecal microbiota transplantation has shown some success in adult patients with UC.^23^ A pilot study in children had shown that faecal microbiota transplantation via enemas was well tolerated and safe, but before its extension into clinical practice, further clinical trial data are needed.^24^

## Acute severe UC

A consensus for managing ASC in children constructed by ECCO has been endorsed by ESPGHAN and the paediatric Porto IBD working group of ESPGHAN as well as the IBD working group of BSPGHAN.^10^ All of these patients require hospital admission. First-line therapy is with intravenous steroids—methylprednisolone at a dosage of 1–1.5 mg/kg/day (up to a maximum of 60 mg/day) given in one or two divided daily doses.^10^ The majority of patients will respond to this treatment.^25^ Of note, 5-ASA medication can cause an acute ‘colitic’ response and should be stopped in ASC and should not be started in patients who are naïve to 5-ASA until they are in the recovery phase to avoid any contribution they may be making to the colitic symptoms.

**Figure 2:**
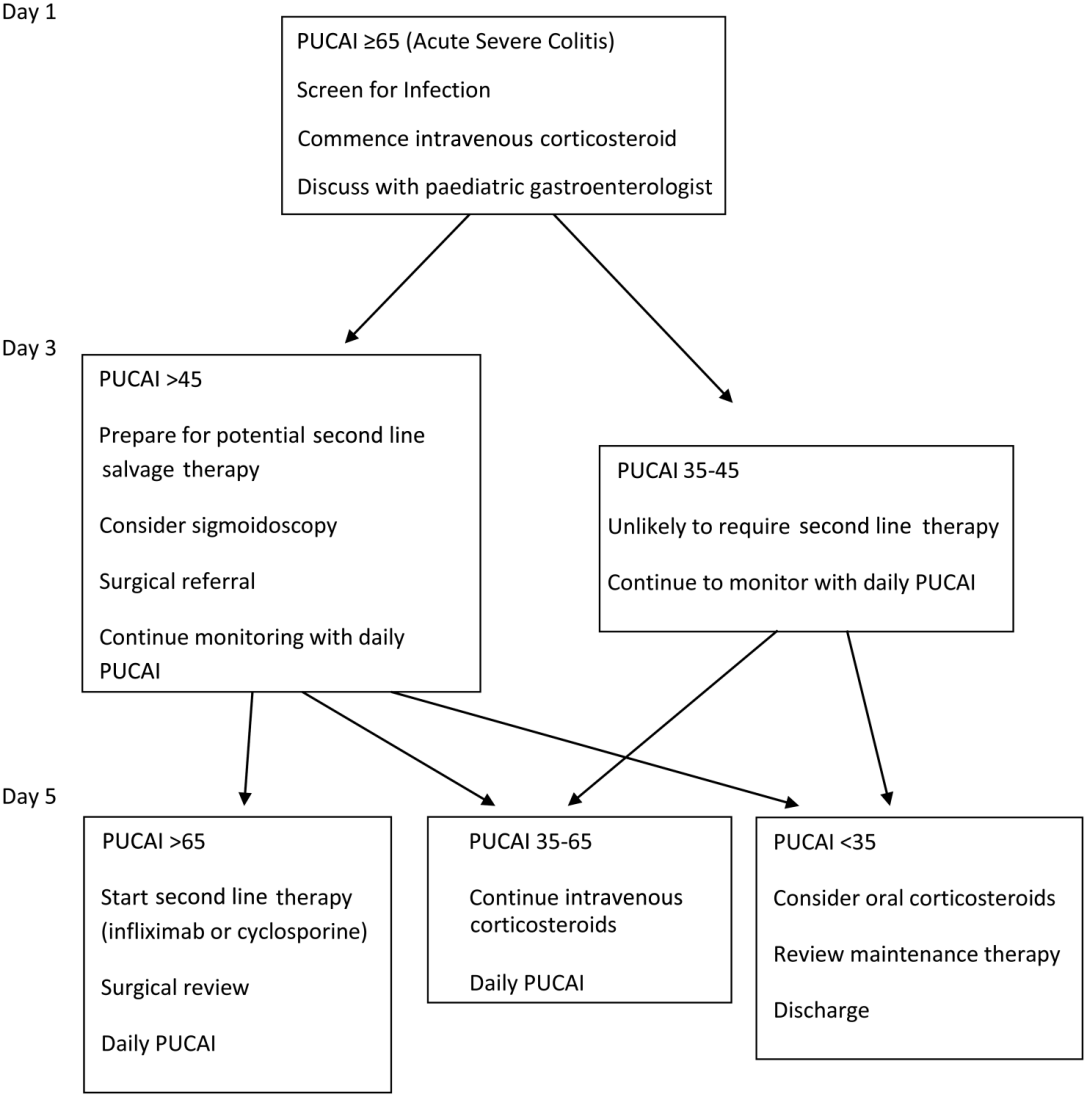
Simplified algorithm of assessment of children with acute severe colitis. This simplified algorithm emphasises the need for daily monitoring with the Paediatric Ulcerative Colitis Activity Index (PUCAI) and timely escalation of therapy in non-responsive cases according to fixed timelines. At day 3 review progress, at day 5 escalate therapy if PUCAI is >65.

Timely introduction of second-line therapy is very important (figure 2). In children, Turner *et al* found that sequential PUCAI scores were effective in identifying those patients requiring a step up in treatment with second-line (salvage) therapy, reducing morbidity plus reducing the need for emergency colectomy.^26^ ^27^ A PUCAI score of >45 on day 3 indicated a likelihood of steroid failure (negative predictive value 94%, positive predictive value 43%), and a PUCAI score of >65 on day 5 indicated the need for salvage therapy with a positive predictive value of 100%, specificity of 94% and negative predictive value of 78%. Second-line salvage therapy, in the absence of toxic megacolon (TMC), involves either calcineurin inhibitors (ciclosporin or tacrolimus) or a biological therapy (infliximab). Traditionally, intravenous ciclosporin was used, but more recently, infliximab usage has increased, avoiding the initial need to monitor drug levels in the acute phase.^25^ Both can act as a bridge to establishing thiopurines. If previous thiopurine therapy has failed, however, infliximab may be the preferred treatment as it offers the option of maintenance in the longer term. Please refer to consensus paper for more detailed information.^10^

Surgery should always be discussed at an early stage with the patient and family depending on individual profiles and concerns. Any patient with ASC unresponsive to steroids on day 3 should have a surgical assessment (if not previously done), and this is mandatory if TMC is suspected. An abdominal X-ray should be taken. Dilatation of the transverse colon >56 mm plus signs of toxicity is diagnostic for TMC, with >40 mm sufficient in children <10 years of age.^28^ Escalating pain in ASC should lead to an assessment to exclude TMC immediately.

## Surgical management of paediatric UC

Surgery is an integral part of the management strategy in patients with UC. The most common surgery that is carried out is a subtotal/total colectomy with a temporary stoma. There is evidence to show that laparoscopic surgery is as safe and effective as open surgery, but has the added benefit of reduced hospital stay, improved patient experience and improved cosmetic outcomes.^29^ ^30^ Restorative surgery can be carried out in 1–3 stage pouch surgery within selected groups of children. Although pouch surgery offers the comfort of not having a stoma for life, there are many concerns that should be discussed to ensure that the child and their parents are aware of the potential risks, which include faecal incontinence, reduced fecundity and erectile dysfunction. Pouchitis is one of the more common problems encountered with antibiotics used as the mainstay of initial treatment.

Surgery is generally perceived as a last resort when all medical options have failed, but this perception and fear of the unknown in conjunction with having a primary stoma makes this a potentially difficult subject to discuss with children and adolescents. However, it is clearly recognised that unplanned/emergency surgery as in ASC along with the feeling of ‘failing’ can have a significant psychological impact on young people and their family, with resultant difficulties accepting the stoma and adapting lifestyles. Therefore, surgery should be discussed in a timely manner with all patients and their family as a potential treatment option in a manner that is appropriate to age and stage of development, particularly if they are steroid dependent and on multiple immunosuppression or in the acute setting.

## Nutritional considerations

Nutritional requirements should not be overlooked in ASC; enteral feeding with regular diet or calorie supplementation is desirable, although bowel rest is not associated with improved outcome even after controlling for disease activity. However, if nutritional requirements cannot be met enterally, parenteral nutrition should be considered early, particularly if surgery appears likely and certainly in the case of TMC.

**Figure 3:**
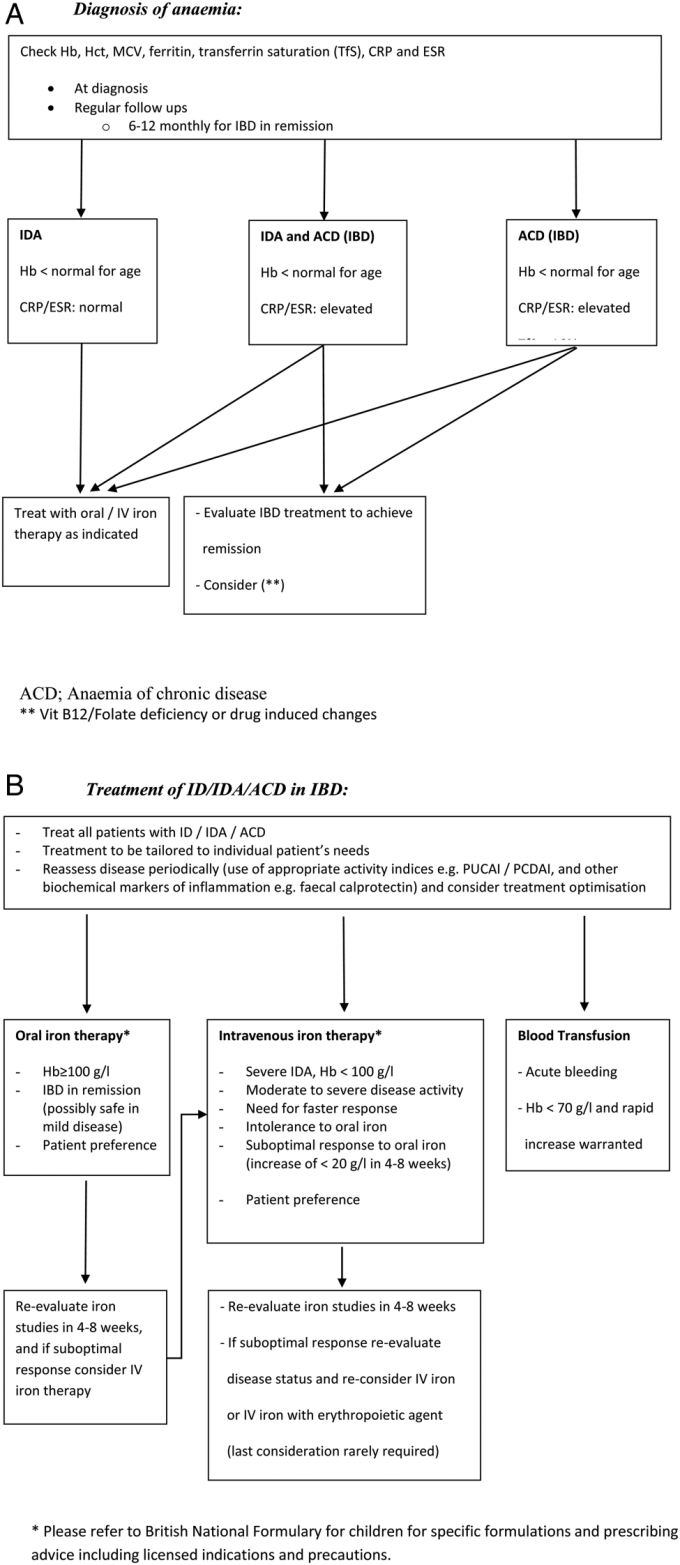
Suggested pathway for diagnosis and treatment of iron-deficiency anaemia. CRP, C reactive protein; ESR, erythrocyte sedimentation rate; Hb, haemoglobin; Hct, haematocrit; IBD, inflammatory bowel disease; ID, iron deficiency; IDA, iron deficiency anaemia; MCV, mean corpuscular volume; PUCAI, Paediatric Ulcerative Colitis Activity Index; PCDAI, paediatric Crohn's disease activity index.

Growth and nutrition are often neglected in patients with UC as it is generally expected that children with UC will reach their full growth potential, which will not be affected if there is good disease control. However, frequent steroid therapy and active inflammation can impact on bone health and growth, which should be actively monitored and treated if identified. Calcium and vitamin D intake should be assessed because of their impact on bone health, especially in patients who have been on prolonged steroids. Iron deficiency was recently identified as a commonly ignored problem in the majority of UK paediatric patients with UC reviewed as part of the UK IBD audit.^25^ We, therefore, have provided a practice-based algorithm to help teams both identify and treat this problem (figure 3).

## Conclusion

The guidelines referenced in this review offer a useful summary for how to manage UC. They highlight the need for accurate diagnosis, early initiation of appropriate therapy and ongoing assessment of disease progress and the side effects of treatment. Care is often centralised, but awareness of who and when to refer for assessment both initially and during relapse (particularly for severe colitis) will remain an important part of the overall management of these children.
